# Cigarette Smoking and Its Hazards in Kidney Transplantation

**DOI:** 10.1155/2017/6213814

**Published:** 2017-07-27

**Authors:** Muhammad Abdul Mabood Khalil, Jackson Tan, Said Khamis, Muhammad AshhadUllah Khalil, Rabeea Azmat, Arslan Rahat Ullah

**Affiliations:** ^1^Diaverum Prince Abdul Majeed Renal Centre, Al Imam Ahmad Ibn Hanbal, Jeddah 21146, Saudi Arabia; ^2^RIPAS Hospital, Bandar Seri Begawan BA1710, Brunei Darussalam; ^3^Khyber Teaching Hospital, Peshawar, Khyber Pakhtunkhwa 25000, Pakistan; ^4^Aga Khan University Hospital, Karachi 74800, Pakistan; ^5^Northwest General Hospital & Research Centre, Peshawar, Khyber Pakhtunkhwa 2500, Pakistan

## Abstract

Cigarette smoking affects many organs. It causes vasoconstriction through activation of sympathetic nervous system which leads to elevation of blood pressure and reduction in glomerular filtration rate and filtration pressure. It also causes thickening of renal arterioles. Cigarette smoking increases the risk of microalbuminuria and accelerates progression of microalbuminuria to macroalbuminuria. Furthermore, it causes rapid loss of glomerular filtration rate in chronic kidney disease patients. After kidney donation, these factors may be injurious to the solitary kidney. Kidney donors with history of cigarette smoking are prone to develop perioperative complications, pneumonia, and wound infection. Postkidney transplantation various stressors including warm and cold ischemia time, delayed graft function, and exposure to calcineurin inhibitors may result in poor graft function. Continuation of cigarette smoking in kidney transplant recipients will add further risk. In this review, we will specifically discuss the effects of cigarette smoking on normal kidneys, live kidney donors, and kidney transplant recipients. This will include adverse effects of cigarette smoking on graft and patient survival, cardiovascular events, rejection, infections, and cancers in kidney transplant recipients. Lastly, the impact of kidney transplantation on behavior and smoking cessation will also be discussed.

## 1. Introduction

Cigarette smoking is common worldwide, despite the numerous deterrent measures that have been put in place over the decades. The number of smokers reported in 2015 was 1.1 billion [1]. Worldwide, tobacco use causes nearly 6 million deaths per year, and current trends show that tobacco use will cause more than 8 million deaths annually by 2030 [2]. On average, smokers die 10 years earlier than nonsmokers [3]. The association of cigarette smoking with cardiovascular diseases, chronic obstructive pulmonary disease, and cancers is well known. Cigarette smoking increases the risk of coronary heart disease and stroke by 2–4 times and that of lung cancer by 25 times [4]. Cigarette smoking also causes chronic obstructive pulmonary disease (COPD) and smokers are 12 to 13 times more likely to die from COPD than nonsmokers [4]. The present review focuses on adverse effects of smoking in normal kidneys, kidney donors, and kidney transplant recipient and effect of kidney transplantation on smoking cessation.

## 2. Effect of Cigarette Smoking on Kidney

**Figure 1:**
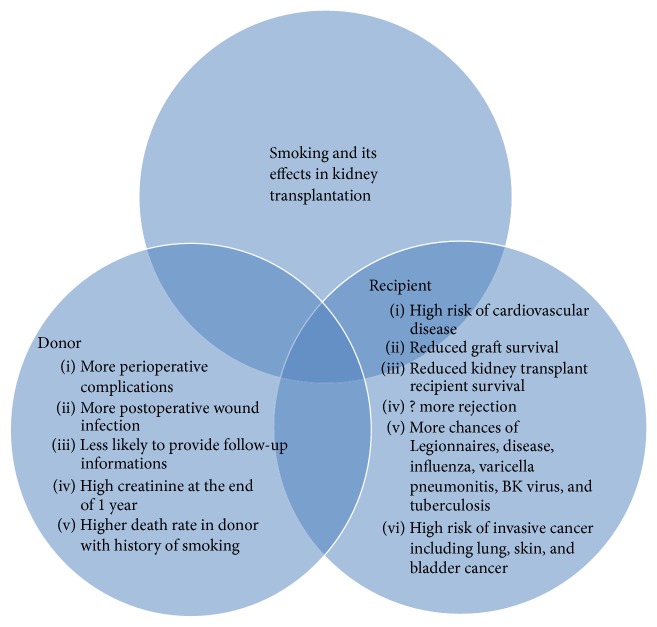
Smoking and its effects in kidney transplantation.

Cigarette smoking can cause acute and chronic effects [5, 6]. Acutely, cigarette smoking increases sympathetic nervous system activity resulting in tachycardia and high blood pressure. Increased sympathetic nervous system activity causes increased catecholamine activity in the circulation. This causes vasoconstriction in the vascular system [7]. Vascular resistance in renovascular bed increases by 11% [7]. This reduces glomerular filtration rate by 15% and filtration fraction by 18%. The chronic effects of cigarette smoking on kidney are less clear. There is evidence that renal plasma flow decreases in chronic smokers and this is accompanied by modest elevation of endothelin. Endothelin through vasoconstriction will induce functional abnormalities. Cigarette smoking has been associated with thickening of renal and myocardial arterioles [8, 9] and has been shown to be an independent predictor of proteinuria [10, 11]. The effect of cigarette smoking in diabetic kidneys has been documented in various studies. Cigarette smoking increases the risk of microalbuminuria in smokers [12]. It also causes rapid progression of microalbuminuria to macroalbuminuria [13] and causes rapid loss of glomerular filtration rate (GFR) leading to rapid progression of diabetic nephropathy [14]. Beside diabetic kidney disease, cigarette smoking has been implicated in nondiabetic kidney diseases. Various studies have shown progression of nondiabetic chronic kidney diseases due to cigarette smoking [15, 16]. Cigarette smoking is deleterious after kidney transplantation.  Figure 1 shows cigarette smoking and its effects in both kidney donors and recipient.

## 3. Cigarette Smoking and Kidney Donors

Kidney donors undergo general anesthesia for donor nephrectomy and are prone to develop complications in the perioperative period. Cigarette smoking causes increased bronchial secretion and impaired mucociliary clearance. It also results in increased carboxyhemoglobin and secondary polycythemia. Stopping cigarette smoking for only 12 hours can greatly reduce carboxyhemoglobin concentrations, improve oxygen content and availability, and reverse negative inotropic and arrhythmic effects [17, 18]. Smokers' polycythemia and blood viscosity reverses within few days while sputum production declines over a period of 6 weeks after smoking cessation [19]. Pneumonia is the third most common infection after urinary tract and wound infection in kidney donors [20]. Smokers have a higher risk of pulmonary and wound infections after surgery than nonsmokers [21]. Based on this data, the Amsterdam Forum Guidelines recommends cessation of cigarette smoking 6 weeks before kidney donation [22].

There is plenty of available data on kidney transplant recipients implicating cigarette smoking in patient and graft survival. However, there is paucity of work on the effects cigarette smoking on kidney donors. A recent study by Segev's and his colleagues through a multivariate analysis adjusting for age, gender, race, systolic BP, and history of hypertension revealed a significantly higher death rate among kidney donors who were smokers [23]. Cigarette smoking in kidney donors is associated with higher rate of perioperative complications [24] and postoperative wound infections [25]. These donors are less likely to provide follow-up information requested by transplant centers [26]. There are mixed results for impact on graft function. Robert et al. found that cigarette smoking in cadaveric kidney donor may be associated with delayed graft function on univariate analysis [27]. But after adjusting for correlated outcome data and controlling for other potential prognostic factors, this relationship became insignificant. In a retrospective analysis by Heldt et al., kidney donors who actively smoke or have a past history of tobacco use were found to have higher serum creatinine at end of 1 year as compared to nonsmokers [28]. Similarly, recipients of kidneys from smokers had higher creatinine and lower GFR [28]. However, Jha et al. found no difference in postoperative complications and graft survival between the two groups [3]. Interestingly though, donor cigarette smoking reduced recipient survival [29].

For live kidney donors, transplantation provides an opportunity to quit cigarette smoking. Keles et al. reported significant drop in cigarette smoking from 47% to 29% after kidney donation [30]. Therefore, kidney donors with history of cigarette smoking should be referred to smoking cessation clinics. More studies are needed to compare the impact of cigarette smoking in kidney donors on donor and recipient survival and graft outcomes.  Table 1 shows summary of various studies done in kidney donors.

## 4. Cigarette Smoking and Kidney Recipient

It is well established that cigarette smoking causes chronic obstructive airway disease, coronary artery disease, and cancer [4]. Cigarette smoking is also associated with increased mortality and graft loss in kidney transplant recipients [31]. In this section, we will review the implications of cigarette smoking on cardiovascular disease, mortality, graft survival, rejection, infections, and cancer in kidney transplant recipients.

### 4.1. Cigarette Smoking and Cardiovascular Diseases in Kidney Transplant Recipient

Cardiovascular disease is the leading cause of death in kidney transplant recipients. Patients usually die with a well-functioning graft due to cardiovascular events. Cigarette smoking can lead to poorer survival of the transplanted kidney, partly due to its role in atherosclerosis, endothelial dysfunction, and vascular disease [32]. Kidney transplant recipients who smoke have a relative risk of 1.9 of having coronary artery disease as reported by Kasiske et al. [33]. Shorter survival has been reported in various studies due to cardiovascular diseases and cigarette smoking has been implicated as one of the contributing factors [34, 35]. Ponticelli et al. reported that cigarette smoking was associated with a higher risk of cardiovascular diseases [36]. The risk of cardiovascular diseases increases with the increasing pack-years of smoking per year. The relative risk for major cardiovascular disease events with cigarette smoking 11 to 25 pack-years at transplant was 1.56, whereas that of cigarette smoking for more than 25 pack-years was 2.14 [37]. Kidney transplant recipients who smoke have higher prevalence of diabetes, hypertension, and dyslipidemia which would predispose them to a higher risk of cardiovascular deaths [38].

In summary, cigarette smokers, therefore, have more cardiovascular events. The risk goes up with increasing number of pack-years. Kidney transplant recipients have more prevalence of other atherogenic risk factors including diabetes, hypertension, and dyslipidemia. Smoking in combination with these risk factors put kidney transplant recipients at risk of more cardiovascular events.

### 4.2. Cigarette Smoking and Graft Survival

Cigarette smoking causes increased sympathetic nervous system activity [5, 6], increases renal arterioles thickness [8, 9], worsens proteinuria [10, 11], and causes rapid loss of glomerular filtration rate in chronic kidney disease [12]. We expect cigarette smoking to cause the same effects in transplanted kidney too. In addition, transplanted kidney undergoes various stressors including warm ischemia, cold ischemia, and delayed graft function and, later on, rejection and exposure to calcineurin inhibitors. Not surprisingly, all these factors may add up and affect long-term graft survival. There is plenty of evidence available that smoking reduces graft survival [33, 34, 39, 40, 42, 44, 45]. Various studies have reported relative risk of 1.30–2.3 [32, 42–44] and hazard ratio of 1.74 to 3.3 for graft loss [29, 39]. Remote past smoking may have little impact on graft survival [39]. It has been shown that quitting cigarette smoking for more than 5 year before transplantation reduce the relative risk of graft failure by 34% [33]. Therefore, smoking cessation advice should be given to all chronic kidney disease patients waiting for transplant to make sure that they stop smoking well ahead of their surgery. Heavy cigarette smoking has been shown to be associated with higher risk of graft failure compared to moderate smoking. Kasiske and Klinger reported that cigarette smoking of more than 25 pack-years at transplantation (compared to cigarette smoking less than 25 pack-years or never having smoked) was associated with a 30% higher risk of graft failure [37]. The effect of cigarette smoking may not be evident earlier on and may appear later. Gombos et al. did not find any difference in serum creatinine at 3 months, but creatinine at 1 year was significantly higher in smokers [40]. Sung et al. reported that pretransplant cigarette smoking had kidney graft survival of 84%, 65%, and 48% at 1, 5, and 10 years, respectively [41], compared with graft survival in nonsmokers of 88%, 78%, and 62% (*P* = 0.007). One may argue that graft loss may be due to death with a functional graft due to smoking. However, death-censored graft survival was also adversely affected by pretransplant smoking in recipients of both cadaveric (*P* = 0.02) and living donor kidneys (*P* = 0.02) [41]. Smoking related chronic obstructive airway disease is common in transplant recipients [42]. Hurst et al. found reduced graft survival even if the patients with chronic obstructive air way disease were excluded [42]. These findings suggest that cigarette smoking has deleterious effect on graft survival irrespective of its effects on mortality or causing chronic obstructive airway disease. However, not all studies demonstrated deleterious effects of cigarette smoking on graft function. Our literature review revealed at least 2 studies which did not show any correlation between cigarette smoking and reduced graft survival [45, 46].  Table 2 summarizes studies done on cigarette smoking and its effects on graft survival.

### 4.3. Cigarette Smoking and Patient Survival in Kidney Transplant Recipient

There are a number of studies that showed increased mortality in kidney transplant recipients due to cigarette smoking [29, 31, 33–35, 39, 42, 45, 48, 49]. Our review of literature showed two meta-analyses on recipient mortality. One meta-analysis, done in solid organ transplantation, showed shorter patient survival and higher odds of mortality in smokers [47]. In another meta-analysis from Iran [31] done in kidney transplant recipients only, the relative risk for death varied between 0.8 and 2.2. Increased mortality has been reported in heavy smokers. Cigarette smoking of more than 25 pack-years at time of transplantation was associated with increased mortality [33]. Luckily, the effect of cigarette smoking on mortality vanishes after 5 years of quitting signifying the importance of continuous efforts to counsel patients to quit cigarette smoking [33]. The effect of cigarette smoking on mortality may appear later in life. Cigarette smoking did not affect mortality during the first year; however, after 1 year, smoking leads to increased mortality [34]. Active smoking has been shown to reduce graft survival as compared to past smoking. However, both active and past smoking have been shown to increase mortality in the recipients [39]. Hurst et al. [42] found that, compared with never smokers, incident cigarette smoking after transplant was also associated with increased risk of death. Donor cigarette smoking is also an important factor in causing mortality along with recipient smoking. In fact, the two together may augment each other's effects. Smoking in combination with other factors leads to higher mortality [29]. History of current and previous smoking in recipients with malignancy is also associated with increased mortality [48]. Similarly, increasing age, diabetes, and hypertension in combination with smoking lead to increased mortality [49]. The effect of smoking on mortality has been enormous and it is considered to be equivalent to diabetes [34]. From this discussion, it emerges that both active and past cigarette smoking adversely affect recipient survival. The effect on mortality is more in heavy smoker. Smoking in combination with increasing age, diabetes, hypertension, and malignancy causes increased mortality. Therefore, all efforts should be done in convincing recipients to quit smoking.  Table 3 summarizes studies done on implication of cigarette smoking on kidney transplant recipient survival.

### 4.4. Cigarette Smoking and Allograft Rejection

Many epidemiologic studies have shown late allograft rejection in kidney, heart, and bone marrow transplant in cigarette smokers [36, 41, 50–55]. The mechanism by which cigarette smoking causes rejection was studied in animal model by Wan et al. [56]. Costimulatory blockade by blocking CD154 induces indoleamine 2,3-dioxygenase activity in pancreatic allograft in mice. This in turn leads to increased survival of the graft. Wan et al. showed that second-hand smoking in mice suppressed mRNA and protein expression of indoleamine 2,3-dioxygenase and shortens allograft survival in a cause-effect manner [56]. The author concluded that their finding was due to cigarette smoking related allograft rejection. Nogueira et al. compared live related kidney transplant recipients between smokers and never smokers [57]. They found that the risk of rejection on or before posttransplant day 10 was much higher (adjusted HR, 1.8; 95% CI, 1.10–2.94; *P* = 0.02) in smokers. These findings are not unanimous as there are other studies that did not show any associations. Sung et al. in their cohort did not find any relation between cigarette smoking and rejection [41]. No relation was found between smoking and rejection by Kheradmand and Shahbazian [38]. An open randomized trial is currently being conducted to determine the effectiveness of carbon monoxide-oximetry and anticigarette smoking counselling in a cohort of kidney transplant patients who smoke [58]. This randomized open label study will assess its effectiveness on cessation of cigarette smoking and will measure various outcomes including rejection. The study may give some insight about association of smoking with rejection.

### 4.5. Cigarette Smoking and Infections in Kidney Transplant Recipient

Cigarette smoking affects the function of white blood cells. Migration and chemotaxis of polymorph nuclear cells decrease in smokers when compared to nonsmokers [59, 60]. Macrophages from smoker lungs have more potent inhibitory effects on lymphocytes resulting in reduced cellular immunity [61]. Cigarette smoking decreases various proinflammatory cytokines such as IL-1, IL-6, tumor necrosis factor *α*, and IL-2 [62–65]. The effect of cigarette smoking on lymphocytes is confusing. There is increase in the ratio of CD4+ to CD8+ lymphocytes in light smokers. This was due to the increase in CD4+ cells in light to moderate smoker who smokes less than 50 packs per year [66–68]. In contrast, there are a decrease CD4+ cells and an increase in CD8+ lymphocytes in heavy smokers (greater than 50 packs per year) [67]. Other studies have reported no difference in the CD4+ and CD8+ lymphocyte counts among moderate smokers [69]. Since CD4^+^ lymphocytes are important for B cell proliferation, decreased levels can potentially result in less antibodies production. Similarly, increase in CD8^+^ cells has been associated with infection and malignancy [70]. Natural killer cells and immunoglobulin (IgA, IgG, and IgM) levels are also reduced in smokers, which will reduce immune threshold further [71–73]. Tobacco use is an independent predictor of all-cause and infection-related mortality in hemodialysis patients [74, 75]. Keeping these factors in mind, kidney transplant recipients with reduced immunity will be at more risk of developing infections.

Not surprisingly, the recipients are prone to multiple infections after transplantation. Legionnaires' disease is common in kidney transplant recipients causing community acquired pneumonia in 1–3% [76] and smoking has been its independent risk factor [77]. The risk of Legionnaires' disease in the general population increases by 121% by cigarette smoking of 1 pack of cigarette daily [78]. We expect that this risk may be even more severe in kidney transplant recipients who have less immunity as compared to general population. Several studies in the general population have shown association of cigarette smoking with influenza infection [79–81]. Similar association was found between cigarette smoking and influenza in kidney transplant recipients [82]. Varicella infections are also common in kidney transplant recipients [83, 84] and it has been shown in studies involving the general population that smokers are at higher risk of contracting varicella pneumonitis resulting in admissions when compared to nonsmokers [85]. Population studies have shown that the risk of varicella pneumonitis increases by 15 folds in smokers [86]. We expect that this will hold true for kidney transplant recipients as well to have more varicella pneumonitis. Tuberculosis is also common in kidney transplant recipients [87, 88] and there is plenty of evidence in general population describing the association of cigarette smoking with tuberculosis [89–92]. Cigarette smoking in combination with BK virus increases risk of bladder cancers in renal transplant recipients [93]. Human papilloma virus is also associated with cigarette smoking. Minkoff et al. [94] reported higher baseline HPV16 and HPV18 DNA load in active smokers. The combination of cigarette smoking with high viral load of human papilloma virus (HPV) in kidney transplant recipients will not only increase the likelihood of infection but also may lead to development of HPV-related tumors.

There is plenty of evidence linking cigarette smoking with various infections in general population. However there is a paucity of good data to confirm this relationship in patients with kidney transplants. An ongoing open label randomized control trial in kidney transplant recipients is under way. Recipient will be followed up for 1 year to assess effectiveness of smoking cessation and the study will measure various variable including infections [58]. This study may provide more evidence for relationship of smoking with infection in the future.

### 4.6. Cigarette Smoking and Cancers in Kidney Transplant Recipient

Immunosuppression in kidney transplant recipients increases risk of cancer [95]. Squamous cell carcinoma is 20 times more common in kidney transplant recipient as compared to the general population [96]. Immunosuppression increases risk of cancer by two mechanisms. Immune surveillance for tumor antigen is reduced resulting in more opportunistic viral infections like cytomegalovirus, human papilloma virus, Epstein-Barr virus, and hepatitis B and C virus. These viruses in turn increase risk of malignancy. It is well known that cigarette smoking increases the risk of cancer in general population [97, 98]. Nonmelanoma skin cancers are one of the major causes of morbidity after organ transplantation [99]. Squamous cell carcinoma is the commonest nonmelanoma cancer of all the cutaneous malignancies [99, 100], with a 65–100-fold increase of incidence in transplant recipients compared to the general population. Sun exposure, sensitivity to ultraviolet radiation, and cigarette smoking have been implicated as etiological factors in squamous cell carcinoma [101]. Human papilloma virus is another risk factor for squamous cell carcinoma and it has been found that active smokers have higher baseline HPV16 and HPV18 DNA load [94]. This can theoretically increase the risk of squamous cell carcinoma. However, in a meta-analysis [47], in solid organ transplantations, posttransplant cigarette smoking was positively correlated with nonskin malignancies (OR 2.58, 95% CI 1.26–5.29). Lymphoproliferative disorders are also common in kidney transplant recipients [102]. Since cigarette smoking has been implicated in follicular lymphoma and Hodgkin's disease in the general population [103, 104], it is possible that it may play an important causative role in the transplant population as well. Human papilloma virus and cervical carcinoma are well documented in transplant recipients [105]. By extension, one can assume that cigarette smoking may have a more pathogenic role in causation of cervical carcinoma in transplant patients.

Kasiske and Klinger showed that cigarette smoking was a risk factor for invasive malignancies [37]. The relative risk increases from 0.97 to 1.99 with increasing number of packs of cigarettes. However, cigarette smoking had no statistically significant effect on cancers other than lung cancer [37]. Cigarette smoking cessation for more than 5 years before transplantation did not reduce the risk of cancer in this study. Nägele et al. [52] in a study done with heart transplant patients reported higher rate of malignancies (7/22 smokers developed cancer, as compared to 4 cancers in 62 nonsmokers, *P* = 0.0001). Increasing age, sun exposure, and cigarette smoking habits have been shown to increase risk of malignancy in various studies in kidney transplant recipients [106–108]. Smoking not only has etiological role but also increases mortality in kidney transplant recipients with malignancy. The mortality risk from malignancy is 40% higher in those who continued cigarette smoking as compared to those who stopped cigarette smoking after transplantation [48]. Cigarette smoking and BK virus have been found as significant etiological factors for bladder tumors in kidney transplant recipients [93]. Bearing these evidences in mind, counselling for cigarette smoking cessation should be advocated to all recipients to reduce risk of long-term complications from malignancies.

## 5. Effect of Kidney Transplantation on Cigarette Smoking Behavior

Kidney transplantation provides an opportunity to both donors and recipients to quit cigarette smoking. Continuation of cigarette smoking causes fatal medical events in both donor and recipient. Kidney donors who are smokers are less likely to provide follow-up information requested by transplant centers [26]. Kidney transplantation is in fact an incentive for patients to stop cigarette smoking. Keles and his colleagues [30] reported that preoperative cigarette smoking status in live donors was 47%. Postoperatively, it reduced significantly to 29%. This could be because of predonation counselling. Other reasons for this could be frequent contacts of the donor in the donor clinic and resulting fear that continuation of cigarette smoking may affect the solitary kidney. Banas et al. [109] reported that 27.6% of smokers stopped cigarette smoking after kidney transplantation. The cessation of cigarette smoking was more prevalent in patients < 55 years of age and females. The cessation of cigarette smoking after transplantation in kidney transplant recipients was greater in magnitude when compared to nicotine patch which was successful only 16.4% of the times at 12 months [109]. This was almost comparable with bupropion plus nicotine patch combination which results in cigarette smoking cessation in 35.5% at 12 months of therapy. Major medical events like myocardial infarction and stroke also increase health consciousness to quit cigarette smoking [52]. Getting a new kidney also transforms patient's life and may increase health consciousness of the recipient to quit cigarette smoking. Therefore, extensive counselling should be done with donors and recipients to encourage smoking cessation and this should be continued throughout subsequent clinic consultations.

## 6. Recommendations

Keeping in view the evidence available for hazards due to cigarette smoking, we highly recommend the following:

1. A detailed history of smoking should be taken from both donor and recipient.
2. Detailed explanation of possible hazards of cigarette smoking for the donor (perioperative complications, postoperative wound infection, and kidney dysfunction in future) should be done.
3. Detailed explanation of possible hazards of cigarette smoking in recipient (increased cardiovascular mortality, decreased graft survival, decreased patient survival, possible rejection, infections, and malignancies) should be explained to the recipient.
4. Those with history of cigarette smoking should be referred to dedicated smoking cessation clinic.
5. Kidney donor should stop smoking at least 6 weeks before the surgery.
6. Smoking cessation advice should be part of chronic kidney disease management before transplantation.
7. All efforts should be put in to ensure smoking cessation in kidney transplant recipients at least 6 weeks before surgery.
8. Transplantation results in cigarette smoking cessation in both recipients and donors. However, to achieve 100% cessation, continuous education and counseling should be provided to both donor and recipient by transplant coordinator and transplant physicians
9. Those who continue to smoke should have regular follow-up in smoking cessation clinic. Use of nicotine, bupropion, or varenicline can be used to quit cigarette smoking. Those using bupropion will need to monitor cyclosporine blood level.

## 7. Conclusion

Cigarette smoking has significant implications for both kidney donors and recipients. Cigarette smoking has been associated with perioperative complications, wound infections, and mortality in transplant recipients. Kidney donation increases health consciousness and decreases cigarette smoking in significant number of donors. However, more work is needed to assess the impact of cigarette smoking on renal function and mortality in kidney donors. Cigarette smoking causes increased cardiovascular events and leads to decreased patient and graft survival. Cigarette smoking may also be associated with rejections but this needs further studies for verifications. Additionally, cigarette smoking is associated with opportunistic infections and malignancies in kidney transplant recipients.

## Figures and Tables

**Table 1:** Summary of previous publications on implication of cigarette smoking in kidney donors.

Number	Author	Journal/year	Conclusion
(1)	Segev et al. [23]	JAMA/2010	Higher death rate in kidney donors with cigarette smoking

(2)	Patel et al. [24]	Transplantation/2008	More perioperative complications

(3)	Mjøen et al. [25]	Transplantation/2009	More postoperative wound infections

(4)	Ommen et al. [26]	Am J Transplant/2011	Less likely to provide follow-up information requested by transplant centers

(5)	Robert et al. [27]	J Crit Care/2010	No impact on delayed graft function

(6)	Heldt et al. [28]	AdvUrol/2011	(1) Active smoker or past history of smoking has high creatinine at end of 1 year as compared to the nonsmoker. (2) Recipients had higher creatinine and lower GFR

(7)	Underwood et al. [29]	Clin Transplant/2014	(1) No difference in postoperative complications (2) No effect on graft survival (3) Reduced recipient survival if donor was a smoker

(8)	Keles et al. [30]	Transplant Proc/2015	Significant drop in cigarette smoking from 47% to 29% after kidney donation

**Table 2:** Summary of previous publications on implication of cigarette smoking on graft survival.

Number	Author	Journal/year	Findings
(1)	Kasiske and Klinger [37]	J Am SocNephrol/2000	Smoking more than 25 pack-years at transplantation (compared to smoking less than 25 pack-years or never having smoked) was associated with a 30% higher risk of graft failure (relative risk 1.30; 95% confidence interval [CI], 1.04 to 1.63; *P* = 0.021). Having quit smoking for more than 5 years before transplantation reduced the relative risk of graft failure by 34% (relative risk 0.66; 95% CI, 0.52 to 0.85; *P*, 0.001)

(2)	Agarwal et al. [39]	Am J Nephrol/2011	(1) Past history of smoking in recipient did not have any impact on graft survival (2) Current smoking had higher risk for graft failure compared to never smoking in renal transplant recipient (hazard ratio, HR = 3.3, 95% CI 1.5–7.1, *P* = 0.002)

(3)	Underwood et al. [29]	Clin Transplant./2014	Recipient smoking reduces graft survival (HR = 1.74, *P* = 0.05)

(4)	Gombos et al. [40]	Transplant Proc. 2010	No difference in creatinine at 3 months, but creatinine at 1 year was significantly higher in smokers

(5)	Sung et al. [41]	Transplantation/2001	Patients who were smokers at the time of pretransplant evaluation had kidney graft survival of 84%, 65%, and 48% at 1, 5, and 10 years, respectively, compared with graft survival in nonsmokers of 88%, 78%, and 62% (*P* = 0.007) Pretransplant smoking adversely affected death-censored graft survival in recipients of cadaveric (*P* = 0.02) and of living donor kidneys (*P* = 0.02). In a multivariate analysis, pretransplant smoking was associated with a relative risk of 2.3 for graft loss

(6)	Hurst et al. [42]	Transplantation/2011	(1) Compared with never smokers, incident smoking after transplant was associated with increased risk of death-censored allograft loss (adjusted hazard ratio [AHR] 1.46 [95% confidence interval {CI}: 1.19–1.79]; *P* < 0.001) (2) In a sensitivity analysis excluding patients with history of chronic obstructive pulmonary disease, similar results were obtained with increased risk of death-censored allograft loss (AHR 1.43 [95% CI: 1.16–1.76]; *P* = 0.001) and death (AHR 2.26 [95% CI: 1.91–2.66]; *P* < 0.001)

(7)	Cosio et al. [34]	Clin Transplant./1999	Cox regression analysis showed significantly shorter graft survival in smokers (*P* = 0.0005)

(8)	Matas et al. [43]	Ann Surg./2001	Pretransplant smoking was an important risk factor of a poorer long-term graft survival among recipients with 1-year graft survival (RR, 2.1)

(9)	Woo et al. [44]	J Nephrol/2002	Cox regression analysis showed that cigarette smoking was associated with graft failure having relative risk of 1.81

(10)	Kheradmand and Shahbazian [38]	Urol J/2005	Pretransplant smoking was significantly associated with reduced overall graft survival (*P* = 0.01), but no correlation between smoking cessation after transplantation with survival graft was found

(11)	Yavuz et al. [45]	Transplant Proc/2004	No significant relationship between pretransplant smoking and educational status (*P* = 0.354); graft loss; and smoking (*P* = 0.129) was found

(12)	Mohamed Ali et al. [46]	Saudi J Kidney Dis Transpl./2009	The mean graft survival in patients who were smokers at the time of pretransplant evaluation was 89.3% compared with 92.5% in the nonsmokers (*P* = 0.347)

**Table 3:** Summary of studies on implications of cigarette smoking on kidney transplant recipient survival.

Number	Author	Journal/year	Conclusion
(1)	Kasiske and Klinger [37]	J Am SocNephrol/2000	The increase in graft failure was due to an increase in deaths (adjusted relative risk 1.42; 95% CI, 1.08 to 1.87; *P* = 0.012)

(2)	Agarwal et al. [39]	Am J Nephrol/2011	Current smoking and past smoking in renal transplant recipient resulted in higher risk for death than never smoking (HR = 2.1, 95% CI 1.1–3.8, *P* = 0.016, and HR = 2.4, 95% CI 1.4–4.0, *P* = 0.001, resp.).

(3)	Underwood et al. [29]	Clin Transplant./2014	Both donor and recipient smoking decreased recipient survival (HR = 1.93, *P* < 0.01 versus HR = 1.74, *P* = 0.048)

(4)	Duerinckx et al. [47]	Transplantation/2016	Shorter patient survival time (OR 0.59, 95% CI 0.44–0.79) and higher odds of mortality (OR 1.74, 95% CI 1.21–2.48) in solid organ transplantation

(5)	Opelz and Döhler [48]	Transplantation/2016	Compared with patients who had never smoked (*n* = 31,462), patients who stopped smoking before transplantation has increased chances of death (HR, 1.1; 95% CI, 1.0–1.2; *P* < 0.001) and a similar risk of death-censored graft failure (HR, 1.0, 95% CI, 1.0–1.1; *P* = 0.19), but a 40% increase in death from malignancy (HR, 1.4; 95% CI, 1.2–1.7; *P* < 0.001)

(6)	Hurst et al. [42]	Transplantation/2011	Compared with never smokers, incident smoking after transplant was associated with increased risk death (AHR [adjusted hazard ratio] 2.32 [95% CI: 1.98–2.72]; *P* < 0.001)

(7)	Arend et al. [49]	Nephrol Dial Transplant/1997	Smoking was associated with increased mortality after >1 year posttransplantation RR (95% CI) 2.2 (1.4–3.7)

(8)	Cosio et al. [34]	Clin Transplant./1999	By Cox regression, patient survival time was significantly shorter in diabetics (*P* < 0.0001), smokers (*P* = 0.0005), and recipients older than 40 yr

(9)	Woo et al. [35]	Journal of Nephrol/2002	Smoking was an independent risk factor for patient survival (hazard ratio, 1.81)

(10)	Yavuz et al. [45]	Transplant Proc/2004	No significant relationship between pretransplant smoking and educational status (*P* = 0.354); graft loss; and smoking (*P* = 0.129) was found
